# Detecting the relationships among multivariate time series using reduced auto-regressive modeling

**DOI:** 10.3389/fnetp.2022.943239

**Published:** 2022-10-07

**Authors:** Toshihiro Tanizawa, Tomomichi Nakamura

**Affiliations:** ^1^ Data Analysis Group, InfoTech, Connected Advanced Development Division, Toyota Motor Corporation, Tokyo, Japan; ^2^ Graduate School of Information Science, University of Hyogo, Kobe, Japan

**Keywords:** multivariate time series, statistical modeling, transfer entropy, model selection, auto-regressive models

## Abstract

An information theoretic reduction of auto-regressive modeling called the Reduced Auto-Regressive (RAR) modeling is applied to several multivariate time series as a method to detect the relationships among the components in the time series. The results are compared with the results of the transfer entropy, one of the common techniques for detecting causal relationships. These common techniques are pairwise by definition and could be inappropriate in detecting the relationships in highly complicated dynamical systems. When the relationships between the dynamics of the components are linear and the time scales in the fluctuations of each component are in the same order of magnitude, the results of the RAR model and the transfer entropy are consistent. When the time series contain components that have large differences in the amplitude and the time scales of fluctuation, however, the transfer entropy fails to detect the correct relationships between the components, while the results of the RAR modeling are still correct. For a highly complicated dynamics such as human brain activity observed by electroencephalography measurements, the results of the transfer entropy are drastically different from those of the RAR modeling.

## 1 Introduction

To understand the dynamical properties of any complicated systems including those in physiology, we have to analyze a set of signals generated by the system under consideration, varying in time and interrelated with each other, which is referred to as multivariate time series. Though it is surely important to understand the time dependence of each component of the time series separately, it is also crucial to detect the directed relationships among the components, in which the structure and functionality of the system are partially embodied. In many cases including those in physiology, however, the system is so complicated that we have no theoretical argument to identify the relationships from the first principle and we have to detect them only from observed data.

There are several common techniques for such detection. Among them, the Granger causality is probably the most classical and well-known [Granger (1969)]. This technique tries to detect causal relationship between two components from the improvement of prediction errors of the one of the two components by including the signals of the other component. Other techniques, such as the Directed Transfer Function [Kamiński and Blinowska (1991); Kamiński et al. (2001)] or Partial Directed Coherence [Baccalá and Sameshima (2001)] are based on the Vector Auto-Regressive (VAR) model with the coefficients transformed into the frequency domain to investigate the spectral properties. Schreiber (2000) has introduced another measure to detect the relationships called transfer entropy by an extension of the concept of mutual information.

An important feature of these techniques is that they are pairwise measures. In other words, these measures are calculated by taking all pairwise combinations out of a set of the components contained in the time series. It is not obvious, however, whether the relationships among components more than three can always be broken into pairwise relationships. For instance, let us consider the case in which two pairs of components, (A, B) and (A, C), are directly related within each pair. Despite that there is no direct relationship between B and C, the pairwise measure would detect a non-zero value of indirect relationship *via* A and we need an appropriately chosen threshold to determine the acceptance of this relationship. Though there are several procedures such as the surrogate data method (Theiler et al. 1992) in choice of the threshold value, it would be preferable if we have a method that enables us to extract the direct relationships from an entire set of components without pairwise break-up and threshold.

When the number of components are large (∼100) , it is clear that using pairwise measures is impractical. Relating to this point, Papana et al. (2021) have recently published a comparative study of various causality measures in the time domain aiming at detecting direct causality in multivariate time series. The main focus of the authors is the detectability of the causality measures of direct relationships among multivariate time series of components as many as 100. The authors thus compare causality measures with various dimension reduction techniques, such as subset regression (Breiman 1995; Yang and Wu 2016), model reduction (Brüggemann 2003; Shojaie and Michailidis 2010; Siggiridou and Kugiumtzis 2016), and non-uniform embedding (Vlachos and Kugiumtzis 2010; Faes et al. 2011; Kugiumtzis 2013).

In this article, we investigate multivariate time series of a moderate number of components up to 10 and show that pairwise measures such as transfer entropy might fail in detecting relationships among components even for time series of this relatively small number of components. As a technique that enables us to extract relationships from an entire set of components without pairwise break-up and threshold, we take the Reduced Auto-Regressive (RAR) modeling firstly proposed by Judd and Mees (1995) and compare the results to those of the transfer entropy proposed by Schreiber (2000), which is one of the commonest pairwise measures in the time domain.

This article is organized as follows. In Section 2, we describe the RAR modeling technique and the transfer entropy after setting the mathematical notations. In Section 3, we apply the RAR modeling technique to two artificial systems, both of which are three-component time series defined by linear equations. The results are compared to the values of transfer entropy and it is shown that the transfer entropy cannot detect correct relationships when the time series contains different time scales in fluctuation, even when the signals are generated by linear equations. In Section 4, we apply the RAR modeling technique to a set of electroencephalography (EEG) data composed of 10 channels and compare the results with those of the transfer entropy. Discussion and Summary are in Section 5.

## 2 Theoretical backgrounds

### 2.1 Multivariate time series

We consider a set of multivariate time series, $X={\left\{x\left(t\right)\right\}}_{t=0}^{N-1}=\left\{x\left(0\right),x\left(1\right),\ldots ,x\left(N-1\right)\right\}$ , where $x\left(t\right)={\left(x_{0}\left(t\right),x_{1}\left(t\right),\ldots ,x_{M-1}\left(t\right)\right)}^{T}$ is a column vector composed from *M* signals generated from a system under consideration at discrete time *t* with equal intervals. The superscript *T* stands for taking the transpose. Throughout this article, we consider multivariate time series observed at an equal time interval and the source that generates the *i*-th signal is referred to as the *i*-th component in this article. In time series, the signals at the present time are related to the signals of at some previous time called “lag”. In this article, we are also interested in the relationships among the components. For example, if the present signal of component *i* is determined by previous values of other components, say, 1, 3, 6, at lag 2, 1, 5, respectively, we expect that there might be a mathematical expression

$$x_{i}\left(t\right)=f\left(x_{1}\left(t-2\right),x_{3}\left(t-1\right),x_{6}\left(t-5\right)\right),$$ (1)

where *f* is a function that determines the relationship, which might be potentially non-linear. It should be emphasized that, in this article, the term “relationship” is used only in this meaning and we do not discriminate whether the relationship is “causal” or “correlational”.

### 2.2 Reduced auto-regressive model

The time series modeling for multivariate time series, ${\left\{x\left(t\right)\right\}}_{t=0}^{N-1}$ , attempts to represent the present state of the time series **x**(*t*) by functions of the past states, $\left\{x\left(t-1\right),x\left(t-2\right),\ldots \;\right\}$ ,

$$x_{i}\left(t\right)=f_{i}\left(x\left(t-1\right),x\left(t-2\right),\ldots ,x\left(t-L\right)\right)\;\;\left(i=0,1,\ldots ,M-1\right),$$ (2)

for each *i*-th component, where we denote the maximum time delay (lag) as *L*. When the underlying dynamics of the system generating the multivariate time series is unknown, choosing an appropriate function form, *f*_*i*_, for each component and an appropriate value of the maximum lag, *L*, in practice are no trivial tasks and necessarily become heuristic. In this article, we limit ourselves to the function form in Eq. 2 to be linear with respect to their arguments. This limitation might be considered as a drawback, since quite a few time series data generated by real-world systems are potentially non-linear. Tanizawa et al. (2018) have shown, however, that, even for the case in which the time series data are non-linearly distorted, the linear modeling technique can identify the built-in periodicities correctly. We thus believe that linear modeling has a rather wide range of applicability if the non-linearity is not so strong as to induce the dynamics to be chaotic and if the relationships and periodicities built in the time series are sufficiently retained. In linear modeling, the value of the *i*-th component at time *t* is represented as

$$x_{i}\left(t\right)=a_{i,0}+\underset{j,k}{\sum }a_{i,j,k}\;x_{j}\left(t-l_{i,j,k}\right)+\varepsilon_{i}\left(t\right)\;\;\;\left(i=0,1,\ldots ,M-1\right),$$ (3)

where *a*_*i*,0_ is the constant term in the modeling of the *i*-th component, which is allowed to vanish and *ɛ*_*i*_(*t*) is a dynamic noise, which is an independently and identically distributed Gaussian random variable with mean zero and finite variance at *t*. Apart from the constant term, the value of the *i*-th component at present time is represented by a linear combination of the values of other components, *x*_*j*_(*t* − *l*_*i*,*j*,*k*_), at previous time with lag *l*_*i*,*j*,*k*_ and parameter *a*_*i*,*j*,*k*_. The subscripts of the lags and the parameters, $\left\{i,j,k\right\}$ , indicate that they appear in the modeling of the *i*-th components with the term of the *j*-th component at the *k*-th lag. If we take all the terms $x_{j}\left(t-l\right)\;\left(j=0,1,\ldots ,M-1;l=1,2,\ldots ,L\right)$ up to the maximum lag *L*, this model is identical to the Vector Auto-Regressive (VAR) model.

Here we take another model, which is an information theoretic reduction of linear models and referred to as the Reduced Auto-Regressive (RAR) model [Judd and Mees (1995; 1998)]. The RAR model extracts a subset of terms that are most relevant for describing the behaviors of the multivariate time series selected by a suitably chosen information criterion.

To be concrete, let us assume that we have a set of observed values of four-component multivariate time series, $\left\{x_{i}\left(0\right),x_{i}\left(1\right),\ldots ,x_{i}\left(N-1\right)\right\}\;\left(i=0,1,2,3\right)$ , to be fitted in the linear form Eq. 3,

$${\hat{x}}_{i}\left(t\right)=a_{i,0}+\overset{3}{\underset{j=0}{\sum }}\underset{k}{\sum }a_{i,j,k}\;x_{j}\left(t-l_{i,j,k}\right)\;\;\;\left(i=0,1,2,3\right).$$ (4)

Here, we represent the value of the model for the *i*-th component at time *t* as ${\hat{x}}_{i}\left(t\right)$ , while the observed value as *x*_*i*_(*t*). The terms *x*_*j*_(*t* − *l*_*i*,*j*,*k*_) included in the model are selected from a “pool of terms”, which is called a “dictionary”. For example, if we take the maximum lag as *L* = 25, the dictionary for the model of the *i*-th component contains 101 terms, which are

$$\begin{array}{ll} & \left\{1,x_{0}\left(t-1\right),\ldots ,x_{0}\left(t-25\right),\right.x_{1}\left(t-1\right),\ldots ,x_{1}\left(t-25\right),\\ & \left.x_{2}\left(t-1\right),\ldots ,x_{2}\left(t-25\right),x_{3}\left(t-1\right),\ldots ,x_{3}\left(t-25\right)\right\} \end{array}$$ (5)

with element 1 for the constant term. From this dictionary, we extract the optimal subset of terms and determine the values of parameters, *a*_*i*0_, *a*_*i*,*j*,*k*_ corresponding to the extracted terms by minimizing a suitably chosen information criterion.

Information criteria have a general form,

$$\left(\text{Number of data}\right)\times \log\left(\text{Mean square prediction error}\right)+\left(\text{Penalty for the number of terms}\right).$$ (6)

The mean square prediction error is the average of the squared norm of the prediction error vector, $e={\left(x_{i}\left(0\right)-{\hat{x}}_{i}\left(0\right),x_{i}\left(1\right)-{\hat{x}}_{i}\left(1\right),\ldots ,x_{i}\left(N-1\right)-{\hat{x}}_{i}\left(N-1\right)\right)}^{T}$ , which represents the difference between the observed values and the values calculated from the model, Eq. 4. Since the observed values inevitably contain dynamical and observational noise, minimizing only the mean square prediction error leads to over-fitting and deteriorate the ability of the model in prediction. Information criteria compensate this deficiency with the penalty for the number of terms, which favors a small number of terms in the model. Among several information criteria proposed in the literature, we take the Description Length (DL) suitably modified by Judd and Mees (1995) as the information criterion in this article. This DL has proven to be effective even in modeling nonlinear dynamics and has fewer approximations than other information criteria (Judd and Mees (1998); Small and Judd (1999)). Assuming that the dynamic noise, *ɛ*_*i*_(*t*), in Eq. 2 is Gaussian, and the parameters, *a*_*i*,0_ and *a*_*i*,*j*,*l*_, are chosen to minimize the sum of squares of the prediction errors, **e** ^*T*^ ⋅**e**, Judd and Mees have shown that the description length is bounded by

$$DL\left(K\right)=\left(\frac{N}{2}-1\right)\ln\frac{e^{T}e}{N}+\left(K-1\right)\left(\frac{1}{2}+\ln\gamma \right)-\overset{K}{\underset{i=1}{\sum }}\ln\delta_{i},$$ (7)

where *N* is the length of the time series to be fitted, *K* is the number of the parameters that take non-zero values (or the model size), and the variables *δ*_*i*_ (*i* = 1, 2, *…*, *k*) can be interpreted as the relative precision to which the parameters are specified. For the details of the variables *δ*_*i*_, see Judd and Mees (1995) and Judd and Mees (1998). The number *γ* is a constant and typically fixed to be *γ* = 32 for choosing a small model size *K*.

To extract the optimal subset to minimize DL(K) from the dictionary of terms, we have to resort a practical selection algorithm, since the exhaustive search is an NP-hard problem when the dictionary contains over a dozen of terms. In this article, we adopt an algorithm proposed by Nakamura et al. (2004). Instead of the exhaustive search, this algorithm begins from identifying the model of the shortest size, *K* = 1, then we look for the term to be added to obtain a smaller value of DL. The model size thus become larger one-by-one until DL ceases to decrease, which is called the bottom-up method. To avoid to be trapped in a local minimum, we proceed a little further to increase the model size, *K*, and then go back to decrease the model size one-by-one until DL ceases to decrease, which is called the top-down method. We repeat these bottom-up and top-down methods until the optimal models in two methods coincide with each other. Nakamura et al. (2004) have proven that this algorithm is able to obtain better models in most cases than other algorithms with reasonable computation time.

A typical result of RAR modeling takes the form

$${\hat{x}}_{0}\left(t\right)=1.34+0.39x_{0}\left(t-1\right)-0.20x_{0}\left(t-3\right)+0.31x_{1}\left(t-4\right)+0.20x_{3}\left(t-7\right),$$ (8)

which includes only the terms, *x*_0_(*t* − 1), *x*_0_(*t* − 3), *x*_1_(*t* − 4), and *x*_3_(*t* − 7), in the dictionary. The RAR model thus includes only terms of relevant components and lags, which is the most important difference between the RAR model and the VAR model. Due to this difference, we are able to identify the directed relationships among components in multivariate time series. For instance, Eq. 8 implies that component *x*_0_ is affected by *x*_1_ and *x*_3_ apart from *x*_0_ itself. It should also be emphasized that there are strong information theoretic arguments to support that the RAR model can detect any periodicities built into given time series [Small and Judd (1999)].

### 2.3 Transfer entropy

Transfer entropy is an information theoretic measure for quantifying the information flow between two univariate time series, which we denote here as $\left\{\ldots ,x\left(0\right),x\left(1\right),\ldots ,x\left(N-1\right),\ldots \;\right\}$ and $\left\{\ldots ,\tilde{x}\left(0\right),\tilde{x}\left(1\right),\ldots ,\tilde{x}\left(N-1\right),\ldots \;\right\}$ . They are not necessarily related to each other. Let us assume that the values *x*(*t*) and $\tilde{x}\left(t\right)$ at each time *t* are independent draws from two discrete stochastic variables, $X=\left(x_{0},\ldots ,x_{i},\ldots ,x_{I-1}\right)$ and $\tilde{X}=\left({\tilde{x}}_{0},\ldots ,{\tilde{x}}_{j},\ldots ,{\tilde{x}}_{J-1}\right)$ , respectively, as the simplest example. It is well known in information theory that the average number of bits needed to optimally encode independent draws from *X* is given by the Shannon entropy $H_{X}=-\sum_{i=0}^{I-1}P\left(x_{i}\right)\log_{2}P\left(x_{i}\right)$ , where *P*(*x*_*i*_) is the probability for *X* = *x*_*i*_. The extra information gain of the state of *X* = *x*_*i*_ by obtaining the state of $\tilde{X}$ is measured by the entropy decrease

$$\Delta H_{X\leftarrow \tilde{X}}\left(x_{i}\right)=-P\left(x_{i}\right)\log_{2}P\left(x_{i}\right)-\overset{J-1}{\underset{j=0}{\sum }}P\left({\tilde{x}}_{j}\right)P\left(x_{i}|{\tilde{x}}_{j}\right)\left(-\log_{2}P\left(x_{i}|{\tilde{x}}_{j}\right)\right)$$ (9)

$$=\overset{J-1}{\underset{j=0}{\sum }}P\left(x_{i},{\tilde{x}}_{j}\right)\log_{2}\frac{P\left(x_{i}|{\tilde{x}}_{j}\right)}{P\left(x_{i}\right)},$$ (10)

where $P\left(x_{i},{\tilde{x}}_{j}\right)$ is the joint probability for $\left(X,\tilde{X}\right)=\left(x_{i},{\tilde{x}}_{j}\right)$ and $P\left(x_{i}|{\tilde{x}}_{j}\right)=P\left(x_{i},{\tilde{x}}_{j}\right)/P\left({\tilde{x}}_{j}\right)$ is the conditional probability for *X* = *x*_*i*_ under the condition of $\tilde{X}={\tilde{x}}_{j}$ . Finally the total information gain of *X* by the knowledge of $\tilde{X}$ is obtained by the summation over *x*_*i*_, which is

$$\Delta H_{X\leftarrow \tilde{X}}=\overset{I-1}{\underset{i=0}{\sum }}\Delta H_{X\leftarrow \tilde{X}}\left(x_{i}\right)=\overset{I-1}{\underset{i=0}{\sum }}\overset{J-1}{\underset{j=0}{\sum }}P\left(x_{i},{\tilde{x}}_{j}\right)\log_{2}\frac{P\left(x_{i}|{\tilde{x}}_{j}\right)}{P\left(x_{i}\right)}.$$ (11)

Noticing that

$$\frac{P\left(x_{i}|{\tilde{x}}_{j}\right)}{P\left(x_{i}\right)}=\frac{P\left(x_{i},{\tilde{x}}_{j}\right)}{P\left(x_{i}\right)P\left({\tilde{x}}_{j}\right)},$$ (12)

we see that the information gain in this simplest case is symmetric with respect to *X* and $\tilde{X}$ and measures the mutual correlation between *X* and $\tilde{X}$ .

Schreiber (2000) extended this concept to the directional information flow between two time series. As time series data have correlation in time direction, the joint probability of signals between different times, *P*(*x*(*t*), *x*(*t*′)) cannot be separated as the product, *P*(*x*(*t*)) ⋅ *P*(*x*(*t*′)). By taking this feature into consideration, Schreiber defined the transfer entropy from $\tilde{X}$ to *X* as the information gain of the time series *X* by obtaining the values of $\tilde{X}$ , which is

$$T_{X\leftarrow \tilde{X}}\left(L,k,l\right)=\overset{N-1}{\underset{t=0}{\sum }}P\left(x\left(t+L\right),x_{t}^{\left(k\right)},{\tilde{x}}_{t}^{\left(l\right)}\right)\;\log_{2}\frac{P\left(x\left(t+L\right)|x_{t}^{\left(k\right)},{\tilde{x}}_{t}^{\left(l\right)}\right)}{P\left(x\left(t+L\right)|x_{t}^{\left(k\right)}\right)},$$ (13)

where

$$x_{t}^{\left(k\right)}=\left\{x\left(t-k+1\right),x\left(t-k+2\right),\ldots ,x\left(t\right)\right\},$$ (14)

$${\tilde{x}}_{t}^{\left(l\right)}=\left\{\tilde{x}\left(t-l+1\right),\tilde{x}\left(t-l+2\right),\ldots ,\tilde{x}\left(t\right)\right\}.$$ (15)

Here we slightly extend the definition by Schreiber to include the time difference *L* that can be a positive integer larger than one to measure the effect of time delay in information flow.

The transfer entropy is non-negative and becomes zero when *X* and $\tilde{X}$ are statistically independent. For the values of *k* and *l*, the value *k* = *l* = 1 is commonly used. In this article, we compare two cases for *k* = *l* = 1 and *k* = *l* = 2 in Section 3. It should be noted that the transfer entropy between two time series is asymmetric in *X* and $\tilde{X}$ , which enables us to determine the directional relationship between these two time series. Another important point to be mentioned is that the transfer entropy is a pairwise quantity by definition. To investigate the directional relationships among multivariate time series whose components are more than three, we should calculate and compare the values of transfer entropy of all pairs in the components of the time series.

## 3 Experiments on artificial linear systems

In this section, we apply the RAR modeling technique to two artificial systems, both of which are represented by linear combinations of the terms of three components with various distinctive lags to investigate the directional relationship among the components and compare the results to the ones obtained from the calculated values of transfer entropy. The difference between these two time series is the time scales of fluctuations of each component. While the time scales of fluctuations of all components in the first system (System 1) are similar, the time scales in the second system (System 2) differ from each other.

### 3.1 System 1: A case with fluctuations in similar time scales

**FIGURE 1:**
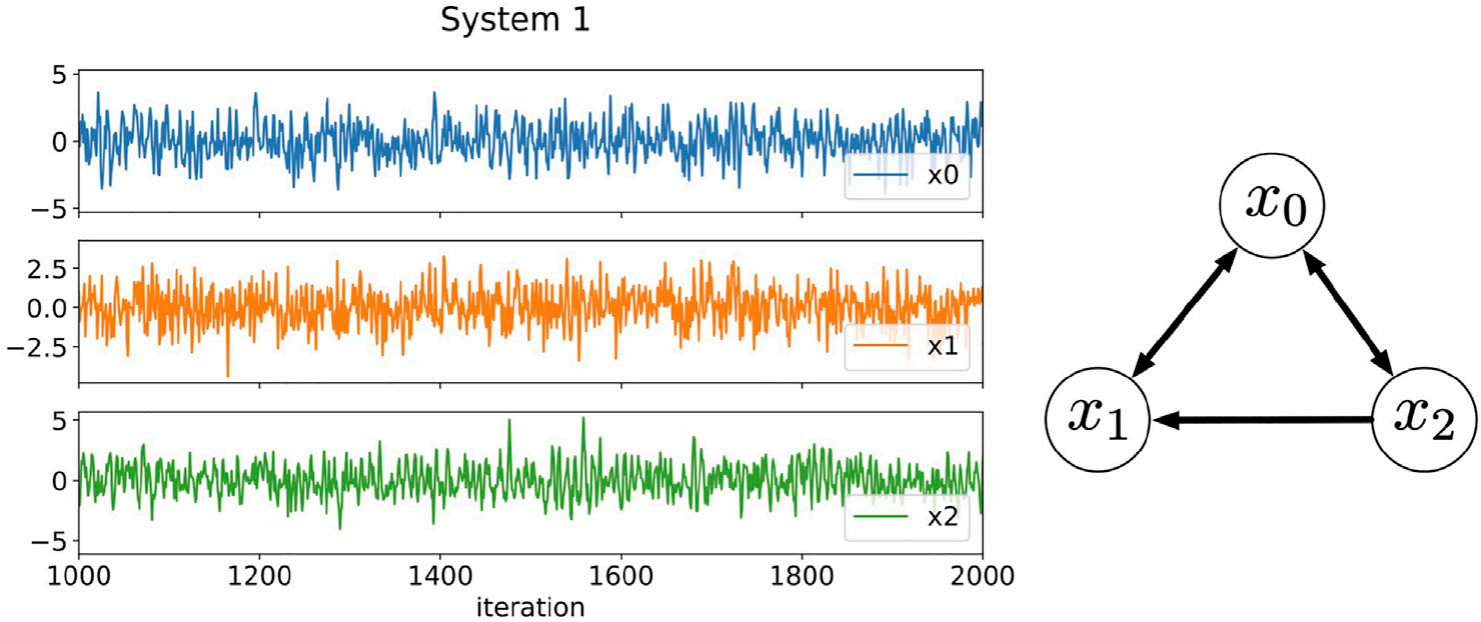
Three-component time series data generated by Eqs. 16–18 and the relationships among the components. The plotted data are a part of results from 1,000 to 2000 iterations. In System 1, the time scales of the fluctuations of each component are in the same order of magnitude.

The time series of System 1 are generated by the following linear equations:

$$x_{0}\left(t\right)=0.4x_{0}\left(t-1\right)-0.2x_{0}\left(t-3\right)+0.3x_{1}\left(t-4\right)+0.2x_{2}\left(t-7\right)+\varepsilon_{0}\left(t\right),$$ (16)

$$x_{1}\left(t\right)=0.2x_{0}\left(t-2\right)+0.3x_{2}\left(t-9\right)+\varepsilon_{1}\left(t\right),$$ (17)

$$x_{2}\left(t\right)=0.2x_{0}\left(t-2\right)+0.5x_{2}\left(t-1\right)-0.3x_{2}\left(t-3\right)+\varepsilon_{2}\left(t\right),$$ (18)

where $\varepsilon_{i}\left(t\right)\;\left(i=0,1,2\right)$ are the dynamic noise that are drawn from independently and identically distributed (IID) Gaussian random variables with mean zero and standard deviation 1.0. This system generates non-divergent signals. The time scales in the fluctuations of each component are in the same order of magnitude, as it can be seen in Figure 1. It should also be noted that component *x*_1_ are generated by other components, *x*_0_ and *x*_2_ and not related to the previous values of *x*_1_ itself. In Figure 1, the relationships among the components are also depicted.

We generate 10000 data points for each component of System 1 after sufficient number of iterations to erase initial value dependence to build the RAR model. In the modeling, we set the maximum time delay *L* = 25. The dictionary contains therefore 76 terms, which are 25 terms for the three components plus one constant term. Having in mind that we build RAR models from electroencephalography data with 1,025 observations in Section 4, we divide these 10,000 data points into 10 intervals each of which contains 1,000 data points and compare the results of RAR modeling corresponding to each divided interval. The results are summarized as

$${\hat{x}}_{0}\left(t\right)=0.41\left(2\right)x_{0}\left(t-1\right)-0.21\left(2\right)x_{0}\left(t-3\right)+0.31\left(4\right)x_{1}\left(t-4\right)+0.20\left(3\right)x_{2}\left(t-7\right),$$ (19)

$${\hat{x}}_{1}\left(t\right)=0.20\left(3\right)x_{0}\left(t-2\right)-0.31\left(2\right)x_{2}\left(t-9\right),$$ (20)

$${\hat{x}}_{2}\left(t\right)=0.20\left(2\right)x_{0}\left(t-2\right)+0.49\left(2\right)x_{2}\left(t-1\right)-0.28\left(3\right)x_{2}\left(t-3\right).$$ (21)

The notation for the values of the parameters such as 0.41(2) represents that the mean value of the parameter of *x*_0_(*t* − 1) over the models for 10 intervals is 0.41 with the standard deviation of 0.02. Notice that all terms included in the definitions, Eqs. 16–18, are recovered with correct values of parameters within appropriate statistical errors and contain no other unnecessary terms.

**FIGURE 2:**
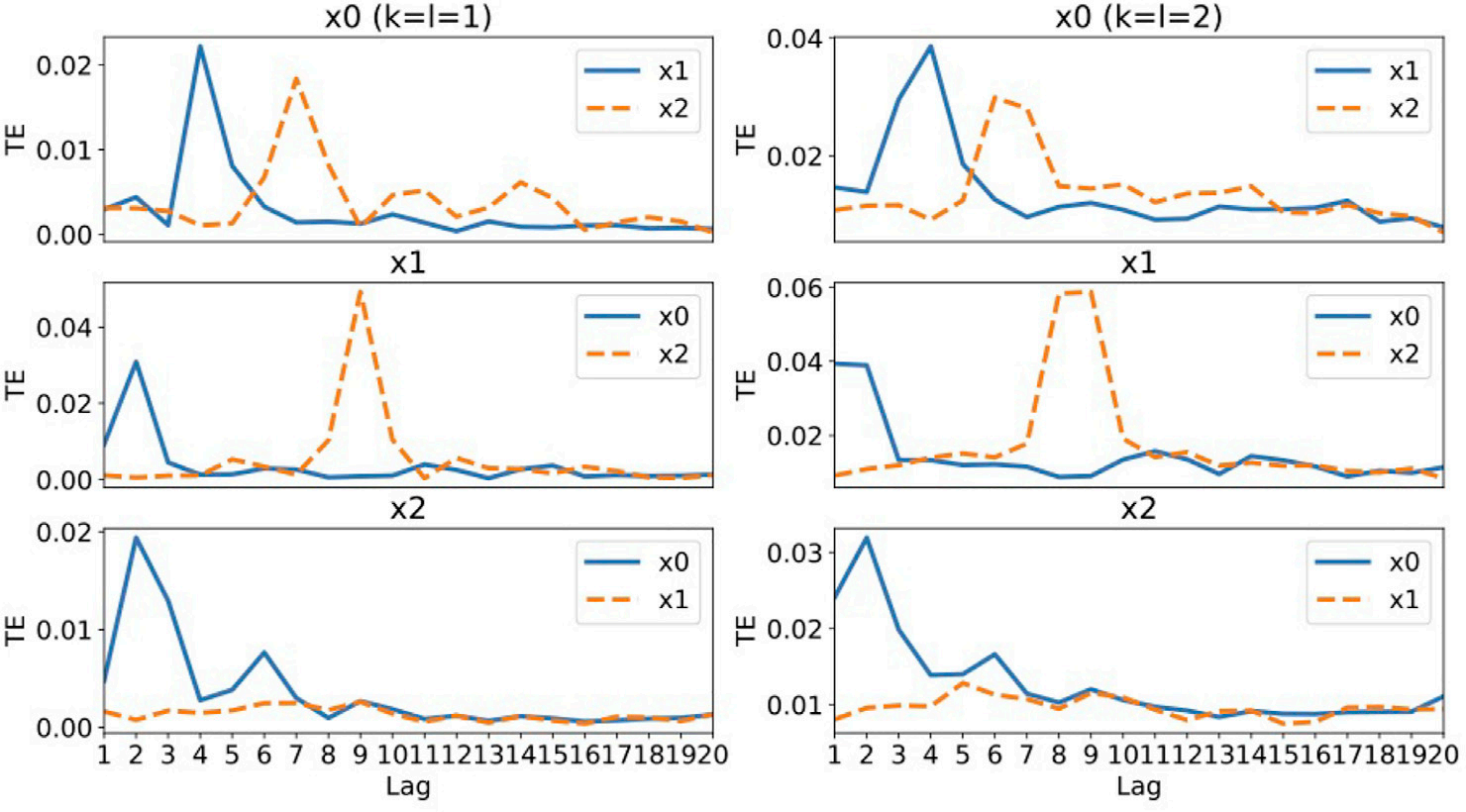
The values of transfer entropy of each component from other components for time delay (lag) up to 20. We plot the values for *k* = *l* = 1 in the left column and the values for *k* = *l* = 2 in the right column for comparison. A large value of transfer entropy indicates that a large amount of information gain exists at the lag from the corresponding components.

Figure 2 shows the values of transfer entropy calculated from the same data as used in the RAR modeling summarized in Eqs. 19–21, though all 10,000 data points for each component are used in this calculation. To see the effect of the values of *k* and *l* in the definition of transfer entropy, Eq. 13, we calculate the values for *k* = *l* = 1, which are most commonly used, and *k* = *l* = 2 for comparison.

Let us examine the results of for *k* = *l* = 1 (the left column of Figure 2). For component *x*_0_, the large values of transfer entropy come from component *x*_1_ at lag 4 and component *x*_3_ at lag 7. Compared to the generator of *x*_0_ defined by Eq. 16, these peaks are consistent with the terms *x*_1_(*t* − 4) and *x*_3_(*t* − 7) in the generator of *x*_0_. For component *x*_1_, peaks appear at lag 2 for component *x*_0_ and at lag 9 for component *x*_2_, which are also consistent with the terms *x*_0_(*t* − 2) and *x*_2_(*t* − 9) in the generator of *x*_1_, Eq. 17. For component *x*_2_, the large value of transfer entropy at lag 2 for component *x*_0_ is consistent with the term *x*_0_(*t* − 2) in Eq. 18, though there is another small peak at lag 6 for component *x*_0_, which does not have any corresponding term in Eq. 18. The values for component *x*_1_ are almost zero, which is reasonable, since component *x*_2_ is independent of *x*_1_. For the results of *k* = *l* = 2 (the right column of Figure 2), the behaviors are almost the same as those of *k* = *l* = 1 except that there appear two consecutive peaks, since the correlation of *x*(*t* + *L*) with *x*(*t*), *x*(*t* − 1), $\hat{x}\left(t\right)$ , and $\hat{x}\left(t-1\right)$ are taken into account for *k* = *l* = 2. As it is also seen later in the results of another artificial system, Figure 4, taking *k* = *l* = 1 would be sufficient for the purpose of identifying the directional relationships among components in time series. For the case of System 1, in which the time scales of the fluctuations of each components are in the same order of magnitude, transfer entropy is able to detect the correct relationships among components in multivariate time series as well as the RAR modeling does.

### 3.2 System 2: A case with fluctuations with different time scales

**FIGURE 3:**
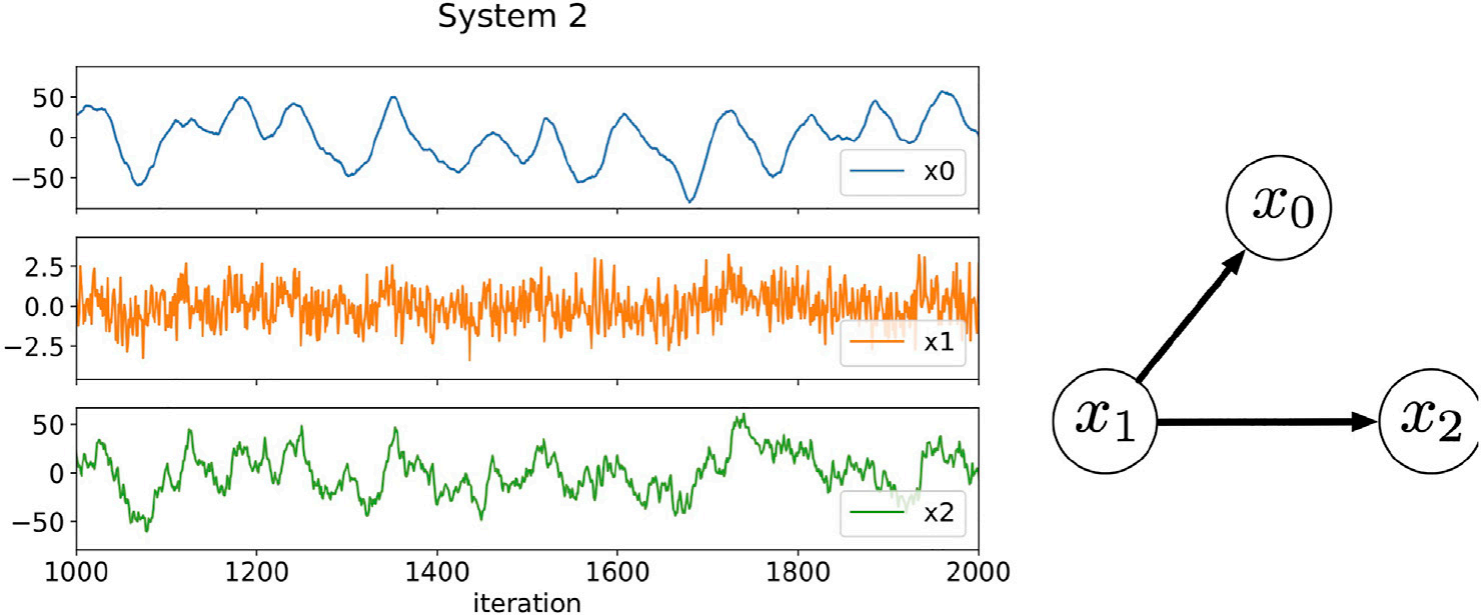
Three-component time series data generated by Eqs. 22–24 and the relationships among the components. The plotted data are a part of results from 1,000 to 2000 iterations. In this artificial system, the time scales in the fluctuations of each component are different: Component *x*_0_ fluctuates slowly, component *x*_1_ fluctuates rapidly, and component *x*_2_ fluctuates intermediately.

The time series of System 2 are generated by the following linear equations:

$$x_{0}\left(t\right)=1.29x_{0}\left(t-1\right)-0.3x_{0}\left(t-4\right)+0.25x_{1}\left(t-3\right)+\varepsilon_{0}\left(t\right),$$ (22)

$$x_{1}\left(t\right)=0.3x_{1}\left(t-1\right)+0.2x_{1}\left(t-6\right)+\varepsilon_{1}\left(t\right),$$ (23)

$$x_{2}\left(t\right)=5.0x_{1}\left(t-3\right)+0.9x_{2}\left(t-1\right)+\varepsilon_{2}\left(t\right),$$ (24)

where $\varepsilon_{i}\left(t\right)\;\left(i=0,1,2\right)$ are the dynamic noise drawn from IID Gaussian random variables with mean zero and standard deviation 1.0 as in System 1. Figure 3 plots the signals generated by these equations and the relationships among the components. The most prominent feature of this system is the differences in the time scale of fluctuation of each component. Component *x*_0_ fluctuates slowly over about 50 iterations, component *x*_1_ fluctuates rapidly in almost every iteration, and component *x*_2_ fluctuates intermediately in time scale between those of *x*_0_ and *x*_1_. It should also be noticed that component *x*_1_, which has the smallest amplitude and is independent of other components, affects components *x*_0_ and *x*_2_. In this regard, System 2 has more complicated characteristics than System 1, even though the dynamics is represented by linear equations.

As in the case of System 1, we generate 10,000 data points for each component of System 2 to build the RAR model, then we divide these 10,000 data points into 10 intervals each of which contains 1,000 data points and compare the corresponding results of RAR modeling. We set the maximum lag as *L* = 25 and use the same dictionary containing 76 terms as used for System 1. The results are summarized as

$${\hat{x}}_{0}\left(t\right)=1.286\left(7\right)x_{0}\left(t-1\right)-0.296\left(8\right)x_{0}\left(t-4\right)+0.25\left(2\right)x_{1}\left(t-3\right),$$ (25)

$${\hat{x}}_{1}\left(t\right)=0.30\left(3\right)x_{1}\left(t-1\right)+0.19\left(2\right)x_{1}\left(t-6\right),$$ (26)

$${\hat{x}}_{2}\left(t\right)=5.01\left(3\right)x_{1}\left(t-3\right)+0.900\left(1\right)x_{2}\left(t-1\right).$$ (27)

As in the case of System 1, all terms and parameters are correctly recovered within reasonable statistical errors for System 2 in spite of the differences in the amplitude and the time scale of fluctuation for each components.

**FIGURE 4:**
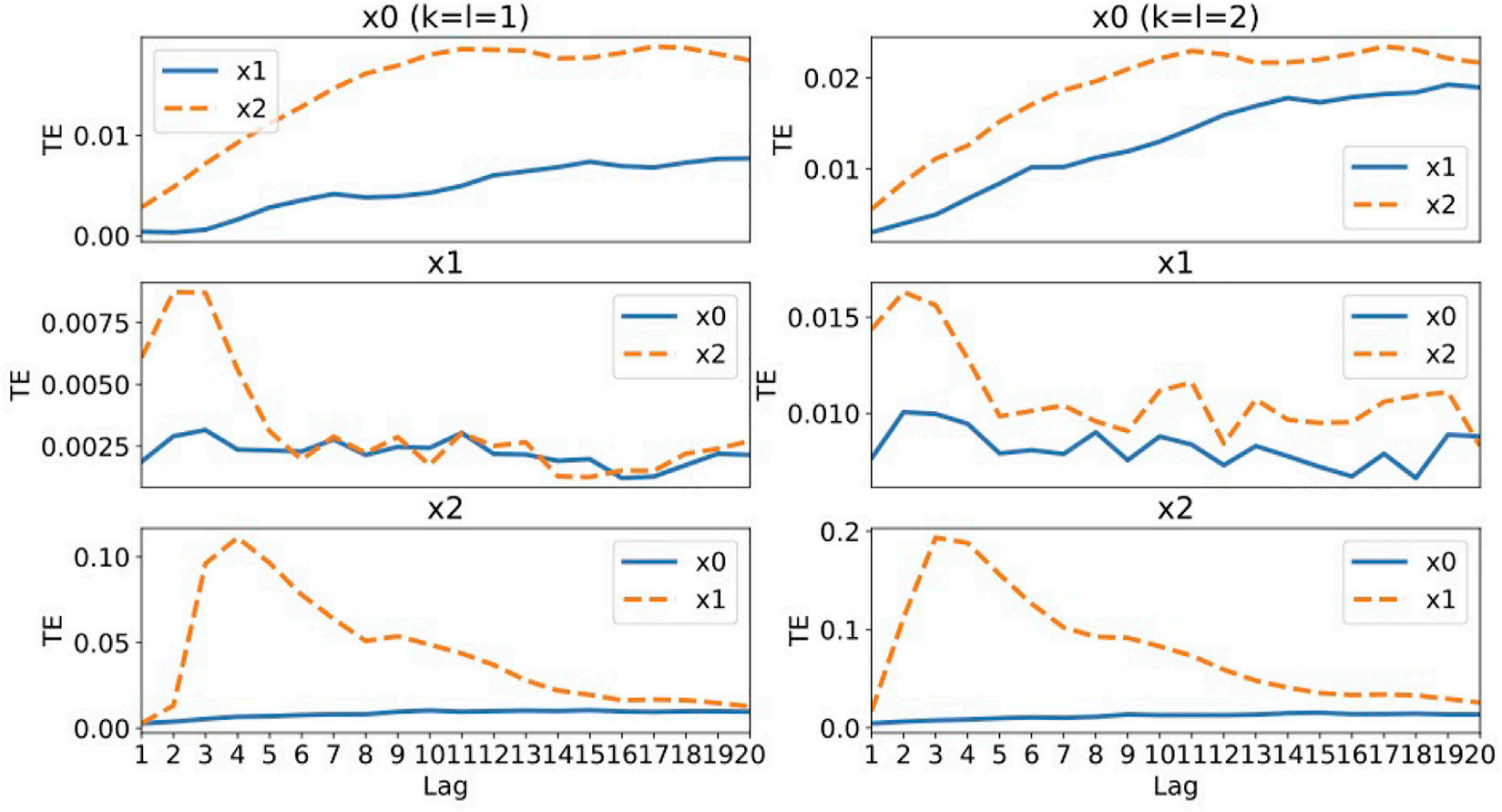
The values of transfer entropy of each component from other components for various values of time delay (lag) up to 20. We plot the values for *k* = *l* = 1 in the left column and the values for *k* = *l* = 2 in the right column for comparison. A large value of transfer entropy indicates that a large amount of information gain exists at the lag from the corresponding components.

Figure 4 shows the values of transfer entropy calculated using all 10,000 data points of the same data as used in the RAR modeling summarized in Eqs. 25–27. As in the case of Systems 1, we calculate the values of transfer entropy for both *k* = *l* = 1 and *k* = *l* = 2 for comparison. First of all, the values of the transfer entropy of component *x*_0_ shows no distinctive peaks, which is remarkably different from those of components *x*_1_ and *x*_2_. Moreover, the values from component *x*_2_ are always larger than those of component *x*_1_, though the generator of *x*_0_ defined by Eq. 22 is independent of component *x*_2_. This deceptive result might be caused by the fact that the amplitudes of components *x*_0_ and *x*_2_ are in the same order. For *x*_1_, the values are very small around 0.0075 and the large values come from *x*_2_ at lags 2 and 3, though there are no such terms in the generator of *x*_1_, Eq. 23. The small values might be related to the fact that component *x*_1_ is independent of other components, though for decisive conclusion for the independence we need to estimate the effect of dynamical and/or observational noise using a method like surrogate generation based approach. For component *x*_2_, the large values of transfer entropy come from *x*_1_ at lags 3 and 4 that might corresponds to the term *x*_1_(*t* − 3) in Eq. 24, though the values of transfer entropy show a long tail after the peak, which might be incompatible with Eq. 24. For the results of *k* = *l* = 2 (the right column of Figure 2), the behaviors are almost the same as those of *k* = *l* = 1. Even if the dynamics is represented by linear equations and the signals contain a small amount of Gaussian noise, the transfer entropy begins to fail in capturing the correct relationships among components for System 2 containing different time scales in fluctuation of each component.

## 4 Results on electroencephalography data

**FIGURE 5:**
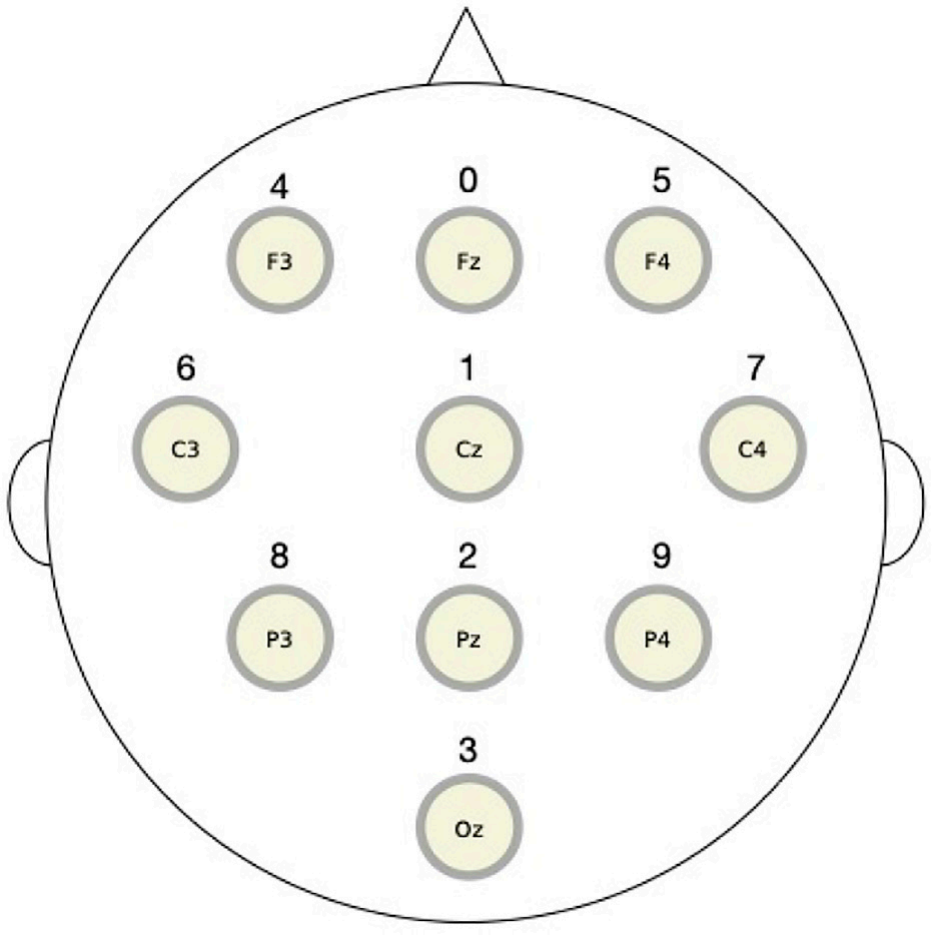
The placement of 10 electrodes in International 10–20 System for electroencephalography measurements. The top (bottom) is the front (back) direction of the head. The digits over the circles representing electrodes are the component numbers used in the RAR modeling.

In this section, we apply the RAR modeling to electroencephalography (EEG) data and compare the results to the values of transfer entropy. The EEG signals used here were recorded from a healthy human adult during resting state with eyes closed in an electrically shielded room and have been analyzed by other methods in Rapp et al. (2005). The data were simultaneously obtained from 10 channels of the unipolar 10–20 Jasper registration scheme and digitized at 1,024 Hz using a twelve-bit digitizer. In Figure 5, we show the placement of 10 electrodes in International 10–20 System. Artifact corrupted records were removed from the analyses. The EEG impedances were less than 5 kΩ. The data were amplified by gain equal to 18,000, and amplifier frequency cut-off settings of 0.03 Hz and 200 Hz were used.

**FIGURE 6:**
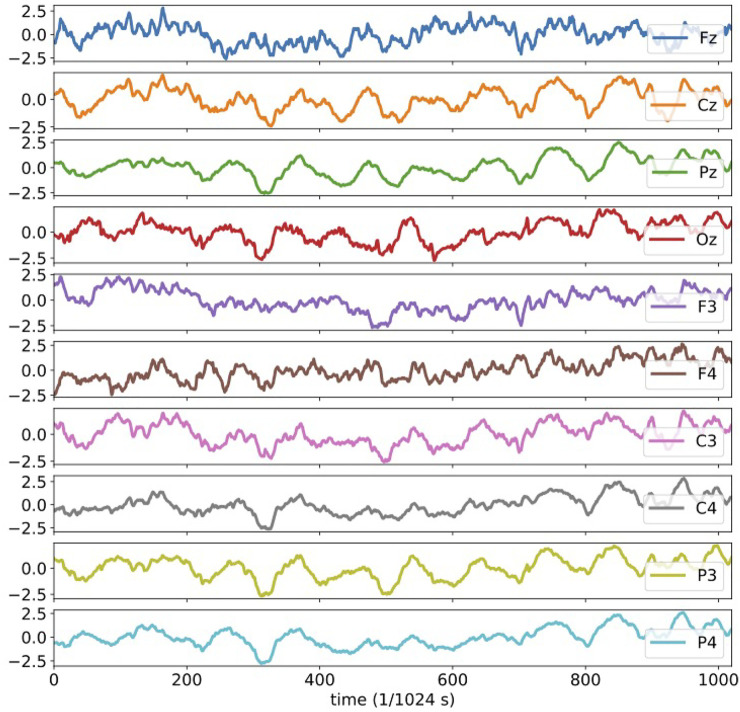
The plots of the 10 channel electroencephalography signals analyzed in the present section. All plotted data are normalized and dimensionless.

The 10 channel electroencephalography signals analyzed here are plotted in Figure 6. It should be noted that the plotted data are all normalized and dimensionless. The activities of human brain are, undoubtedly, highly complicated and non-linear by nature. We should therefore be careful whether there might be suitable interval and duration of time for the dynamics to be approximated in linear forms. Tanizawa et al. (2018) have shown that the RAR modeling is able to detect correct relationships among components even for dynamical systems represented by non-linear differential equations such as the Rössler system. Since there is no explicit description of the results of RAR modeling of the EEG data in Tanizawa et al. (2018), we rebuild the RAR models for these 10 EEG time series, expecting the models contain correct information about the relationships among the components. We use 1,025 data points (1 s) for each component (channel) to build multivariate RAR models. We set the maximum lag as *L* = 25 and the dictionary contains therefore 251 terms, which are 25 terms for the 10 components with one constant term. We show the result for the component Fz explicitly, which is

$${\hat{x}}_{\text{Fz}}\left(t\right)=0.96x_{\text{Fz}}\left(t-1\right)+0.46x_{\text{Cz}}\left(t-1\right)-0.43x_{\text{Cz}}\left(t-2\right)-0.091x_{\text{F}3}\left(t-5\right)+0.17x_{\text{F}3}\left(t-13\right)-0.095x_{\text{F}3}\left(t-20\right).$$ (28)

From this model, component Fz is influenced by component Cz at lag 1 and 2 and component F3 at lag 5, 13, and 20. In Figures 11, 12 of Appendix, we show the behaviors of simulated signals generated from the RAR models and their power spectral densities.

**FIGURE 7:**
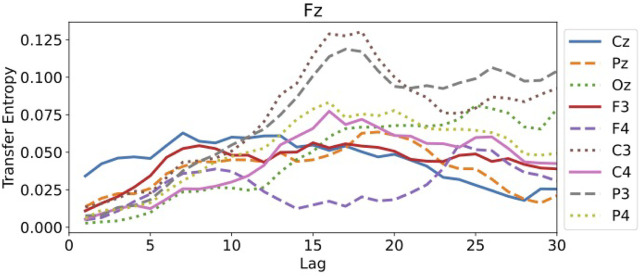
The values of transfer entropy of component Fz from other components with respect to the lags up to 30. All values are in the same order of magnitude and do not show distinct peaks.

**FIGURE 8:**
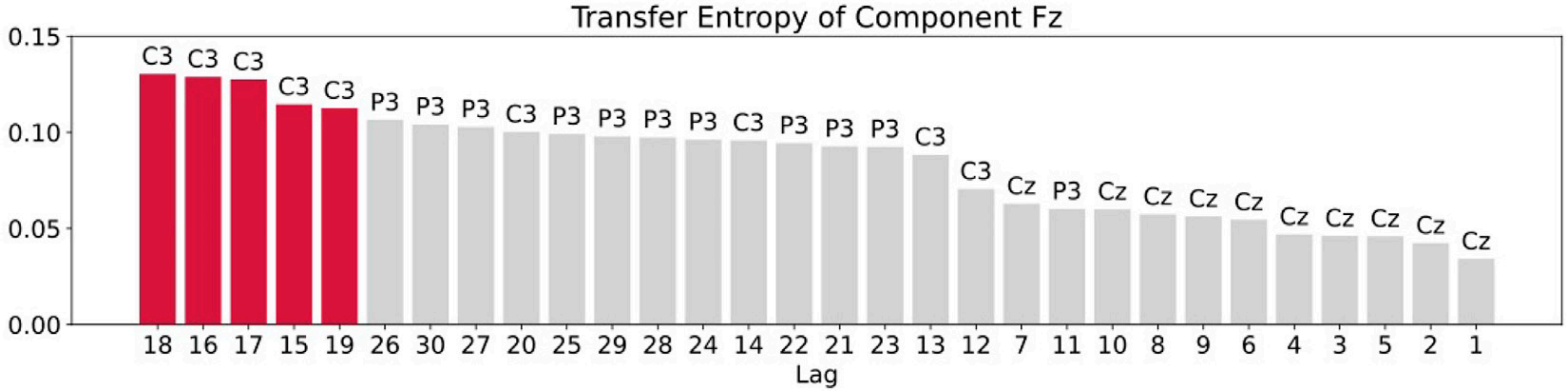
Plot of the components and the maximum values of transfer entropy of component Fz for each lag up to 30 sorted in the descending order of the values of transfer entropy. The red bars are the top five values of transfer entropy. For component Fz, all top five values come from component C3.

For the transfer entropy, we use the same data points as those used in RAR model building and calculate the information gain from the correlation in the signals between all pairs of component Fz and each of other channels up to the maximum lag 30. According to the analysis described in Section 3, we set *k* = *l* = 1 in calculating transfer entropy. The results of the calculation is plotted in Figure 7. From this plot, we see that all values of transfer entropy are in the same order of magnitude and show no distinct peaks that suggest important components and lags. We also plot in Figure 8 the components and the maximum values of transfer entropy of component Fz for each lag up to 30 sorted in the descending order of the values of transfer entropy. According to the calculations of transfer entropy for component Fz, the information gain from component C3 is the largest. The component C3, however, does not appear in the RAR model of component Fz in Eq. 28. The values of transfer entropy for other components show similar behaviors.

**FIGURE 9:**
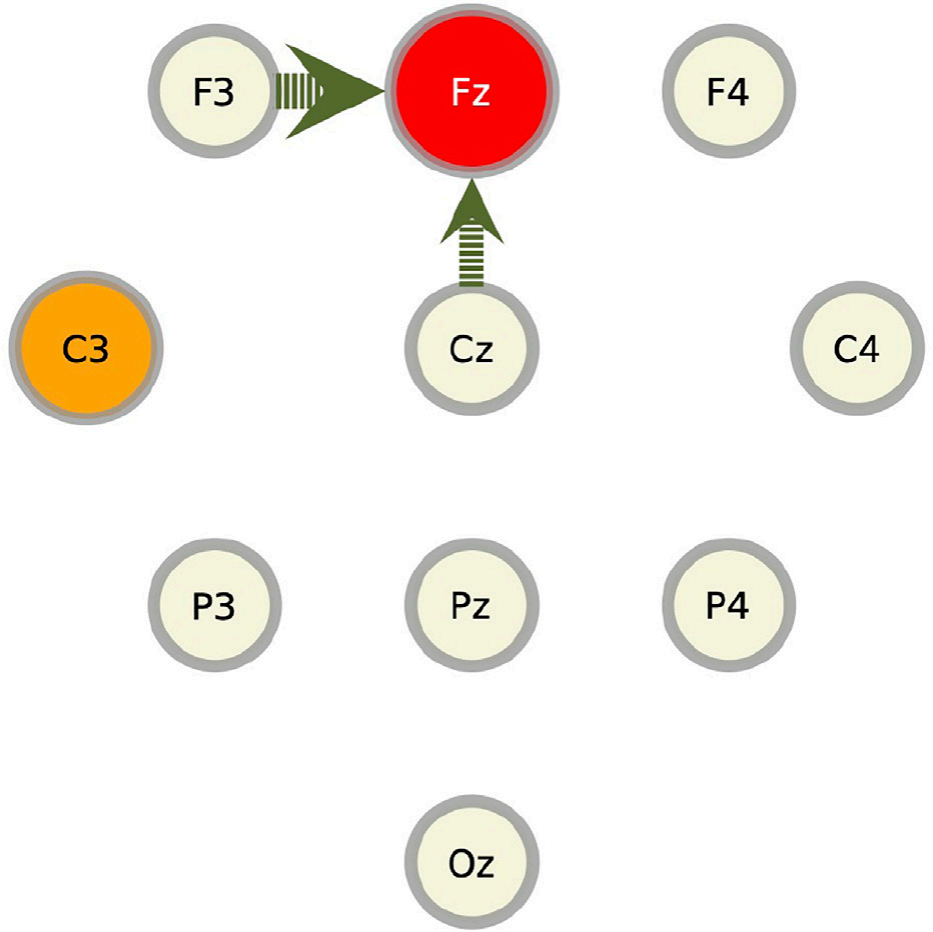
Pictorial summary of the results of the RAR model and the transfer entropy for component Fz. The target component Fz is represented by the red circle. The circles from which the arrows emanate, which are Cz and F3, represent the components contained in the RAR model with the width of the arrows being proportional to the number of appearance of the component in the RAR model. For the case of component Fz, component Cz appears two times and component F3 appears three times. The orange circles represent the components that give the top 5 values of transfer entropy for each lag, which is only C3.

We summarize these results for component Fz in Figure 9. In this figure, the target component against which the RAR model and the transfer entropy are calculated (in this case, Fz), is represented by a red slightly large circle. The circles from which the arrows emanate (in this case, Cz and F3), represent the components contained in the RAR model with the width of the arrows being proportional to the number of appearance of the component in the RAR model. In this case, component Cz appears two times and component F3 appears three times. The orange circles represent the components that give the top 5 values of transfer entropy for each lag. In this case, all top 5 values only come from component C3 (See Figure 8). From this figure, we also see the spatial information of the components included in the RAR model and the components that gives large values of transfer entropy.

**FIGURE 10:**
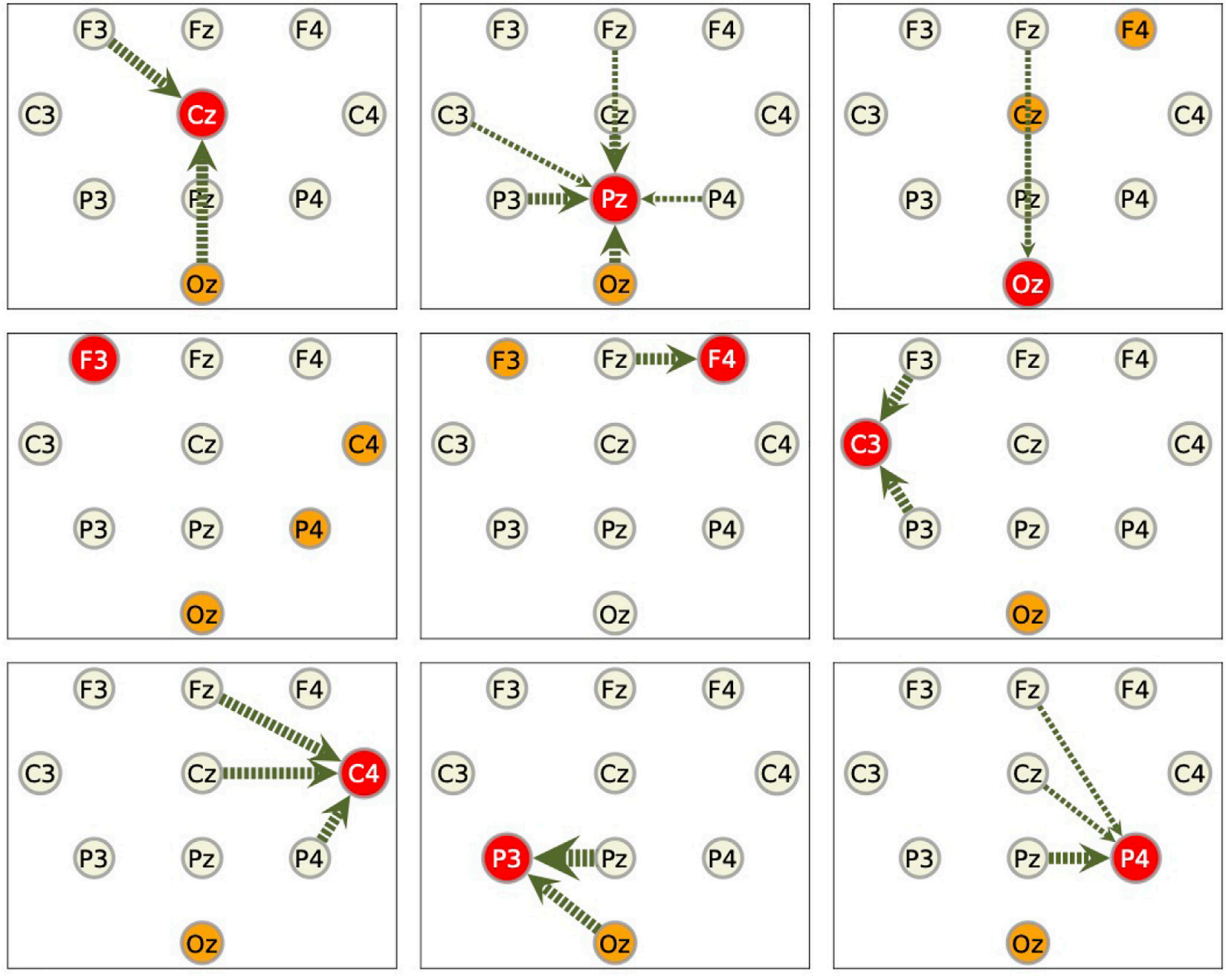
Summarized results for other components. Red circles represent the target components against which the RAR models are built. The arrows are the directed relationships indicated by the corresponding RAR models. The orange circles are the components that gives large values of transfer entropy to the target nodes. See the caption of Figure 9 for the details. Since the RAR model of component F3 contains only terms of F3 itself, there are no arrows in the picture for F3.

**FIGURE 11:**
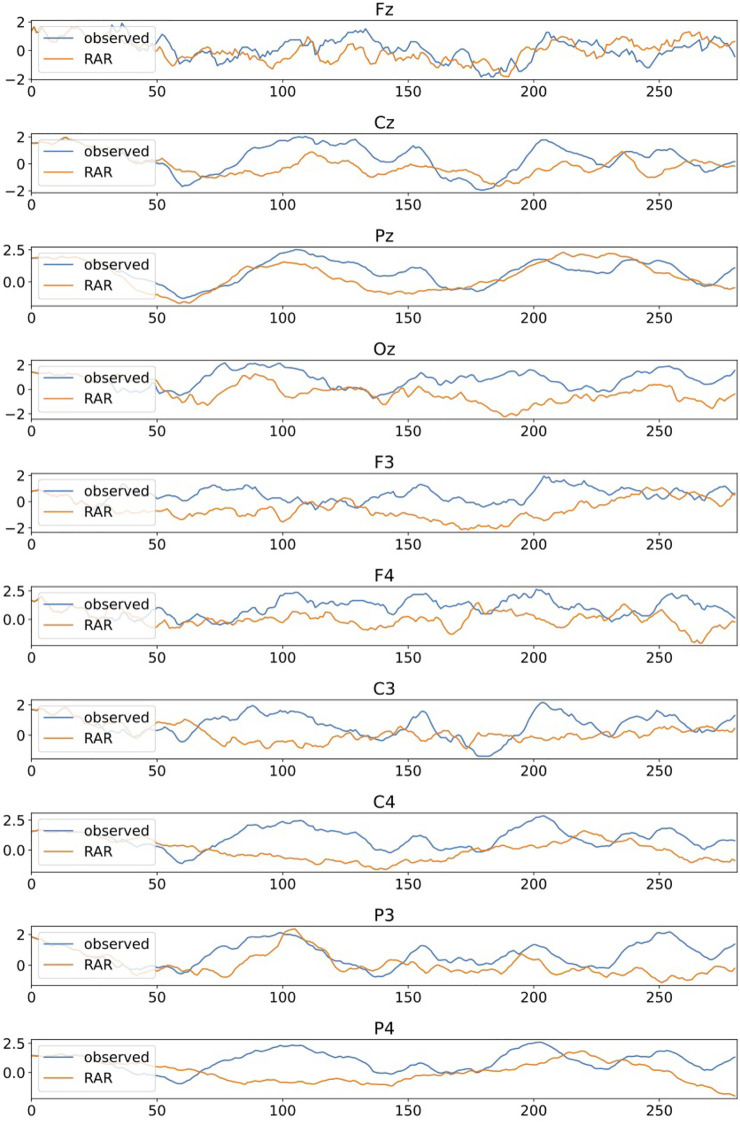
Comparative plots of the observed EEG signals and the signals generated by the RAR models, which are constructed from the first 769 observations of each channel. The observed EEG signals are the last 281 (= 25 + 256) signals of each channel and the RAR signals are generated by the corresponding RAR models with 25 observed signals prior to the last 256 signals as initial values. Simulated signals also contain Gaussian random numbers with mean 0 and standard deviations determined from the fitting errors of each channels in RAR modeling as dynamic noise.

**FIGURE 12:**
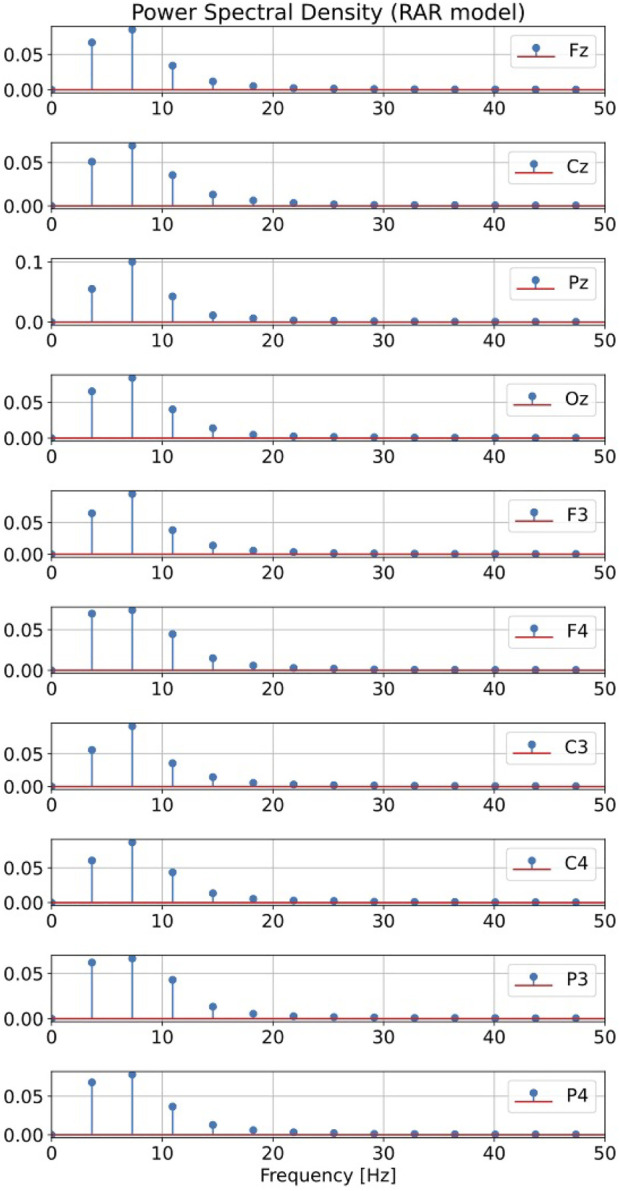
Plots of the power spectral densities of simulated signals from the RAR models. Plotted values are the averages of the power spectral densities over 100 independent runs of simulation. Significant contributions come from frequencies up to about 20 Hz.

As for the other nine components, we show only the summarized results in Figure 10. Generally speaking, the components that give large values of transfer entropy are not related to the components included in the RAR models. It is also to be noticed that the component Oz, which is placed at the back of the head, appears frequently as the component of large transfer entropy values, though it is not likely the outcome of direct influence of this component on the target components.

## 5 Discussion and summary

For two artificial linear systems described in Section 3, the results of the transfer entropy are consistent with those of the RAR modeling, if the values and the time scales in fluctuation of the signals are in the same order of magnitude (System 1). In this case the dynamics of the components are well separated and pairwise methods such as the transfer entropy work well. If the time series contain components whose values and time scale of fluctuation are significantly different from each other (System 2), however, the transfer entropy begins to fail in detecting correct relationships among components, while the RAR modeling is still able to give the correct relationships.

For the application to EEG data in Section 4, the relationships indicated by the results of transfer entropy are drastically different from those indicated by the RAR modeling. Though, within our knowledge, there are no decisive research work in the literature in this regard, we think it is partially because of the insufficiency of pairwise measures for detecting relationships among components that potentially contain various time scales in dynamics for those seen in brain activity. In contrast, it is known that the RAR modeling can detect correct relationships even when the underlying system is non-linear (Tanizawa et al. (2018)). We understand that it would be a controversial issue whether EEG data can be representable by linear models or not. Even in a case in which that the dynamics is represented by a linear system, however, transfer entropy might fail in detecting the correct relationships among the components in multivariate time series, if they contain several time scales in different orders of magnitude. Though we do not claim that the relationships detected by the RAR modeling technique are always correct, detecting the relationships among components in multivariate time series by RAR modeling could be a promising technique with a wide range of applicability.

In summary, we have applied the RAR modeling technique to several multivariate time series as a method to detect the relationships among the components in the time series and compared the results with those of a pairwise measure, transfer entropy in this article. When the relationships between the dynamics of the components are linear and the time scales in the fluctuation of each component are in the same order of magnitude, the results of the RAR model and the transfer entropy are consistent. When the time series contain components that have large differences in the amplitude and the time scales of fluctuation, however, the transfer entropy fails to capture the correct relationships between the components, while the results of the RAR modeling are still correct. For a highly complicated dynamics such as human brain activity observed by electroencephalography measurements, the results of the transfer entropy are drastically different from those of the RAR modeling.

## Data Availability

The original contributions presented in the study are included in the article/Supplementary Material; further inquiries can be directed to the corresponding author.

## Appendix

In this Appendix, we show the behaviors of simulated EEG signals generated by the RAR models and their power spectral densities in the frequency domain. Here the RAR models are constructed from the first 769 observations of each EEG channels to compare the simulated signals to the last 256 observations.

Figure 11 are the plots of simulated signals generated by the RAR models with the last 281 observed EEG signals for comparison. The RAR signals are generated with 25 observed signals prior to the last 256 signals as initial values and contain Gaussian random numbers with mean 0 and standard deviations determined from the fitting errors of each channels in RAR modeling as dynamic noise. Though the observed signals and the simulated ones are not identical because of the dynamic noise, the behaviors seem to be quite similar.

Figure 12 are the plots of the power spectral densities of simulated signals from the RAR models. Plotted values are the averages of the power spectral densities over 100 independent runs of simulation. Significant contributions come from frequencies up to about 20 Hz that correspond to the region of *α* waves.
